# Evaluation of *Helicobacter pylori* eradication and drug therapy in patients with functional dyspepsia

**DOI:** 10.3892/etm.2013.1109

**Published:** 2013-05-13

**Authors:** WEIDONG ZHAO, XIAOQIN ZHONG, XINYING ZHUANG, HONGMEI JI, XINXIN LI, ANQING LI, RUICAI WANG, JIANYOU ZHU, YANQING LI

**Affiliations:** 1 Department of Gastroenterology, Qilu Hospital, Shandong University, Jinan, Shandong 250012;; 2 Departments of Gastroenterology, Shandong 255400, P.R. China; 3 Pathology, People’s Hospital of Linzi District, Zibo, Shandong 255400, P.R. China

**Keywords:** functional dyspepsia, eosinophils, *Helicobacter pylori*, symptom improvement

## Abstract

The aim of this study was to assess the effect of *Helicobactor pylori (H. pylori)* infection and drug therapy on functional dyspepsia (FD) symptoms and gastrointestinal eosinophil count. In this study, 215 continuous FD patients fulfilling Rome III criteria were enrolled. The patients were divided into a *H. pylori*-positive group and a *H. pylori*-negative group. The *H. pylori*-positive group was divided into *H. pylori*-eradicated and *H. pylori*-uneradicated groups following *H. pylori*-eradication treatment, and the *H. pylori*-negative group was randomly divided into esomeprazole and teprenone treatment groups. The symptom scores of the esomeprazole group were significantly lower compared with those of the teprenone group at week 6 but not at baseline and week 2. Compared with the *H. pylori*-uneradicated group, eosinophil counts in the antrum and body were significantly reduced in the *H. pylori*-eradicated group at week 6. The number of gastric eosinophil clusters was significantly higher in the *H. pylori*-positive group than in the *H. pylori*-negative group. Eradication was associated with gastric eosinophil counts but did not affect duodenal eosinophil levels. Neither esomeprazole nor teprenone treatments reduced eosinophil levels in the stomach and duodenum of *H. pylori*-negative patients.

## Introduction

Functional dyspepsia (FD) is a functional syndrome that originates in the gastroduodenal region (1). FD, one of the most prevalent functional gastrointestinal disorders (FGIDs), has prevalence rates of 11.0–29.2% worldwide (2) and reduces quality of life significantly (3–5). Although symptom criteria have been proposed for FD, there remain cases where diagnosis is performed by a method of exclusion, owing to a lack of diagnostic pathology (6,7). Delayed gastric emptying, impaired proximal gastric accommodation to a meal, gastric hypersensitivity to distension, abnormal duodenojejunal motility, psychological disturbance and *Helicobactor pylori* (*H. pylori*) infection have been proposed to be associated with FD in several studies (8–12), however, the pathogenesis of FD remains unclear to date.

Studies have demonstrated that the number of duodenal mucosal eosinophils significantly increases in individuals with FD compared with controls and the degree of eosinophilic infiltration is also correlated with early satiety, which indicates that changes in the number of duodenal eosinophils may be an underlying feature of FD (13,14). In addition, other studies identified that immune activation of duodenal eosinophilia is closely associated with FD, while the increase of gastric eosinophilic granulocytes is not associated with FD (15,16). Eosinophils degranulate and release a variety of substances following activation and help mast cells release substances to stimulate neurons, which leads to contraction of smooth muscle and results in symptoms, including abdominal pain or meal-related symptoms (17). The presence of increased numbers of gastrointestinal eosinophils may serve as a useful biomarker of FD and these potent effector cells may prove to be therapeutic targets in the treatment of these diseases (13).

The role of *H. pylori* in FD remains a controversy. One study indicated that the eradication of *H. pylori* has modest but clear benefits for patients with FD (18). Conversely, another meta-analysis provided little support for the use of *H. pylori* eradication therapy in patients with non-ulcer dyspepsia (19).

Currently, treatment of FD remains challenging (6,7). Observations from clinical practice show that <60% of patients with FD had symptomatic improvement following drug therapy, which is often incomplete (20). This is likely due to the fact that FD is a heterogeneous disease. In general, the approach to treating patients with FD based on their main symptom is practical and effective. Proton-pump inhibitors (PPIs), prokinetics, *H. pylori* eradication and antidepressant drugs are the common choices for the treatment of FD (21).

Although previous studies have investigated the role of *H. pylori* in FD, they did not focus on the correlation between gastroduodenal eosinophil levels and *H. pylori* clearance or drug therapy. The present study was designed to evaluate *H. pylori* eradication treatment in the symptomatic response of patients with FD and to determine whether *H. pylori* eradication and drug therapy affect gastroduodenal eosinophil numbers. In particular, we aimed to discover whether there is a correlation between symptom improvement and the change in gastroduodenal eosinophil numbers following treatment.

## Patients and methods

### Patients

Adult FD patients (aged 18–70 years) fulfilling Rome III criteria were recruited into the study (22). Dyspepsia was defined as epigastric pain, epigastric burning, postprandial fullness and early satiation. All patients had no history of surgery and anaphylactic disease and did not receive antacids, antibiotics, prokinetic drugs or non-steroidal anti-inflammatory drugs during the 4 weeks before the study. Liver and renal function tests, blood glucose, electrolytes, abdominal B ultrasound and upper gastrointestinal tract endoscopy examination were performed for all patients to exclude metabolic and organic diseases. The subjects were required to have no evidence of peptic ulcer disease or gastroesophageal reflux disease with or without esophagitis, malignancy and pancreaticobiliary disease. All subjects signed informed consent forms prior to entering the study. Ethical approval for the study was obtained from the Medical Ethics Committee of Qilu Hospital, Shandong University.

### Abdominal symptom questionnaire

The self-administered abdominal symptom questionnaire assessed symptoms from the upper and lower part of the abdomen over the preceding 3 months (13). A standardized procedure for the administration of the questionnaire at three time points (week 0 as baseline, initial diagnosis and gastroscopy; week 2, end of drug therapy; and week 6, six weeks later after baseline) was conducted. The questionnaire included the following abdominal symptoms: epigastric pain, heartburn, early satiety, postprandial fullness, belching, nausea, vomiting, retching, eructation, anorexia, abdominal distension, epigastric discomfort (noisy), dysphagia and retrosternal pain.

Patients were contacted by telephone and referral to determine clinical symptoms at various time points and the clinical symptoms were marked. In the 5-point Likert table (23), the degrees of the abdominal symptoms (asymptomatic, mild, moderate, severe and very severe) were recorded as 0, 1, 2, 3 and 4 points, respectively, and the scores of four main symptoms (epigastric pain, epigastric burning, postprandial fullness and early satiation) were accumulated for each patient.

### Gastroscopy, biopsy and the ^14^C-urea breath test

Gastroscopy and the ^14^C-urea breath test (Young-heart Medical Appliance Equipment Co., Ltd., Tongcheng, China) were performed for all patients at recruitment. Gastroscopy of all patients was performed by two physicians who were unaware of the symptoms of the subjects before and during endoscopy. At endoscopy, biopsy specimens were collected from the following sites: body (lesser curvature and middle of greater curvature), antrum (lesser curvature and greater curvature), duodenal bulb (D1) and descending part of the duodenum (D2); two specimens were collected from each site. At week 6, certain patients were reexamined by gastroscopy and biopsy and the *H. pylori*-positive patients received the ^14^C-urea breath test again.

### H&E staining and silver staining

Biopsy specimens were fixed in formalin and routinely processed into paraffin wax sections. Sections were cut at 3 *μ*m and stained with hematoxylin and eosin (H&E) and Warthin-Starry stains. *H. pylori* was detected as either positive or negative according to whether the bacteria was observed by Warthin-Starry staining.

The specimens were assessed by two pathologists blinded to the case-control status independently. For each subject, eosinophil counts were obtained from body, antrum, D1 and D2 in five high-power fields (HPF) selected randomly (magnification, ×40). The sum of eosinophils over the 5-field counts were then calculated in each subject. The non-overlapping HPF eosinophil count ≥10 was set as eosinophil cluster positive (13).

### Treatment for FD

The patients were divided into a *H. pylori*-positive and *H. pylori*-negative group according to the results of Warthin-Starry staining and the ^14^C urea breath test. In general, patients in the *H. pylori*-positive group were positive in the Warthin-Starry staining assay and ^14^C urea breath test and patients in the *H. pylori*-negative group were negative in the two examinations. Patients in the *H. pylori*-positive group received *H. pylori* eradication treatment (a quadruple therapy, including 20 mg esomeprazole magnesium tablets, 1 g amoxicillin tablets, 0.5 g clarithromycin dispersible tablets and 0.6 g bismuth potassium citrate, each twice a day for 2 weeks). After 6 weeks, the patients were divided into group A (*H. pylori*-eradicated group) and group B (*H. pylori*-uneradicated group) according to the clearance of *H. pylori* confirmed by the 14C urea breath test.

**Figure 1.**
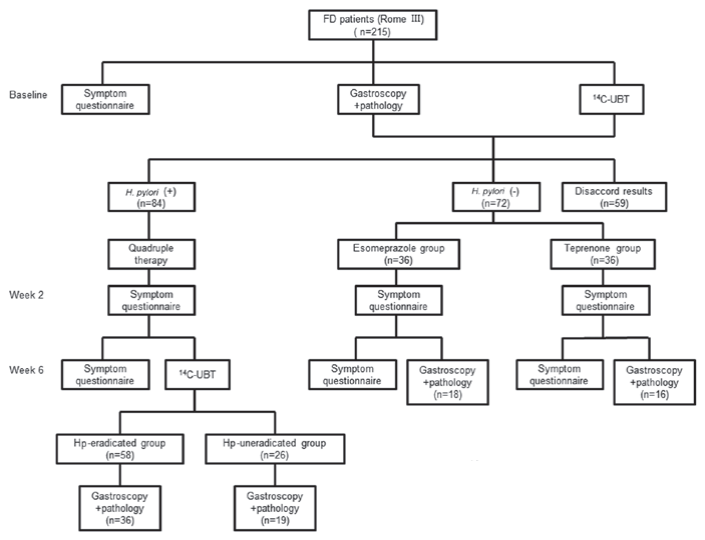
Flow chart of the study. FD, functional dyspepsia; UBT, urea breath test; Hp, *Helicobactor pylori.*

Subjects from the *H. pylori-*negative group were randomly divided into two groups according to a random number table. Group C received 20 mg esomeprazole twice a day (esomeprazole group) and group D received 50 mg teprenone three times a day (teprenone group) for two weeks (Fig. 1).

### Statistical analysis

All data were analyzed by SPSS 20.0 statistical software package (SPSS, Inc., Chicago, IL, USA) and expressed as mean ± standard deviation (SD). The counts of eosinophils and symptom scores were analyzed using single sample t-test among the groups and each time point. The eosinophil cluster rate was examined using χ^2^ analysis. P<0.05 was considered to indicate a statistically significant difference.

## Results

### Patient population

From March 2011 to August 2012, a total of 215 patients (mean age, 54.2 years, 64% female) were enrolled in the study. Of these, 84 patients were positive in the Warthin-Starry stain and ^14^C urea breath test; 72 patients were negative in the Warthin-Starry stain and ^14^C urea breath test; 19 patients were positive in the Warthin-Starry stain but negative in the ^14^C-urea breath test; and 17 patients were negative in the Warthin-Starry stain but positive in the ^14^C-urea breath test. Twelve patients were lost to follow-up and 11 patients were excluded owing to adverse reactions, including unbearable diarrhea and stomach discomfort (6 in the *H. pylori*-positive group, 3 in the teprenone group and 2 in the esomeprazole group). As shown in Fig. 1, the numbers of patients in group A, B, C and D were 58, 26, 36 and 36, respectively, and the numbers of subjects who received gastroscopy at week 6 were 36, 19, 18 and 16, respectively, in the four groups. In the *H. pylori*-positive group, the successful *H. pylori* eradication rate was 69.0% (58/84).

### Upper gastrointestinal pathology

**Figure 2.**
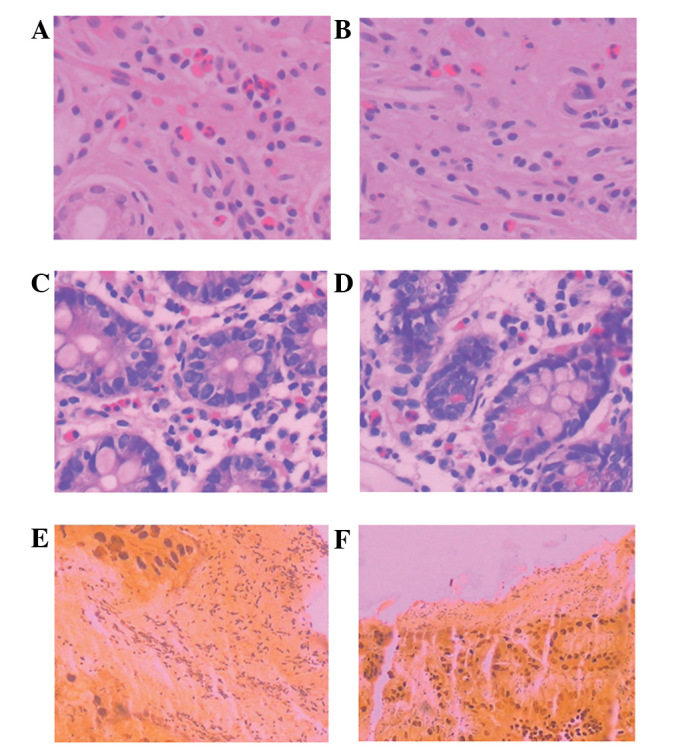
Hematoxylin and eosin (H&E) and Warthin-Starry staining of biopsy specimens from functional dyspepsia (FD) patients. (A and B) H&E staining of a body biopsy from an FD patient, showing typical eosinophil morphology with a bi-lobed nucleus and eosinophilic cytoplasm. (C and D) H&E staining of a descending part of duodenum (D2) biopsy from an FD patient showing clusters of eosinophils in the lamina propria adjacent to glands. (E and F) Warthin-Starry staining of an antrum biopsy showing *H. pylori* stained black while the background was light golden. Magnification, ×40.

The pathology of the FD patients was analyzed by H&E and Warthin-Starry staining of biopsy specimens. Histology by H&E staining in the body and descending part of duodenum (D2) of all patients revealed normal morphology or mild inflammation (Fig. 2A–D). No active duodenitis, visible intestinal parasites or cancer was observed in all subjects. Gastric specimens from the *H. pylori*-positive group processed by Warthin-Starry staining are shown in Fig. 2E and F; *H. pylori* was stained black while the background was light golden.

### H. pylori infection and gastrointestinal symptoms

There was no significant difference in symptom scores between the *H. pylori*-positive group and *H. pylori*-negative group at baseline (Table I). Moreover, in the *H. pylori*-positive group before and after eradication treatment, the symptom scores of the *H. pylori*-eradicated group were overall not significantly different compared with those of the *H. pylori*-uneradicated group at baseline, week 2 and week 6 (Table II). These data suggest that *H. pylori* infection is not associated with the symptoms of FD and *H. pylori* eradication is not necessary when treating this disorder.

### Effect of drug therapy on symptom improvement

To investigate the improvement of infection following various treatments, symptom scores at baseline, week 2 and week 6 after treatment were analyzed. The symptom scores of the four groups were all improved at week 2 after treatment compared with baseline (Table II). The same result was also obtained in the *H. pylori*-eradicated group, *H. pylori*-uneradicated group and esomeprazole group between baseline and week 6, but not in the teprenone group (Table II). At baseline, the symptom scores of the esomeprazole group and the teprenone group were almost the same; however, the symptom scores of the esomeprazole group were significantly improved compared with those of the teprenone group at week 6 (Table II).

### H. pylori infection and gastroduodenal eosinophil counts

At the baseline level, the eosinophil counts in the body and antrum were significantly increased in the *H. pylori*-positive group compared with the *H. pylori*-negative group (Table I, Fig. 2). However, in the duodenum (including the duodenal bulb and descending part of the duodenum), the *H. pylori*-positive group did not demonstrate a marked increase in eosinophil count compared with the *H. pylori*-negative group (Table I). These results demonstrate that *H. pylori* infection upregulates gastric eosinophil counts but has no effect on duodenal eosinophil counts in patients with FD.

### Effect of drug therapy on gastroduodenal eosinophil counts

**Figure 3.**
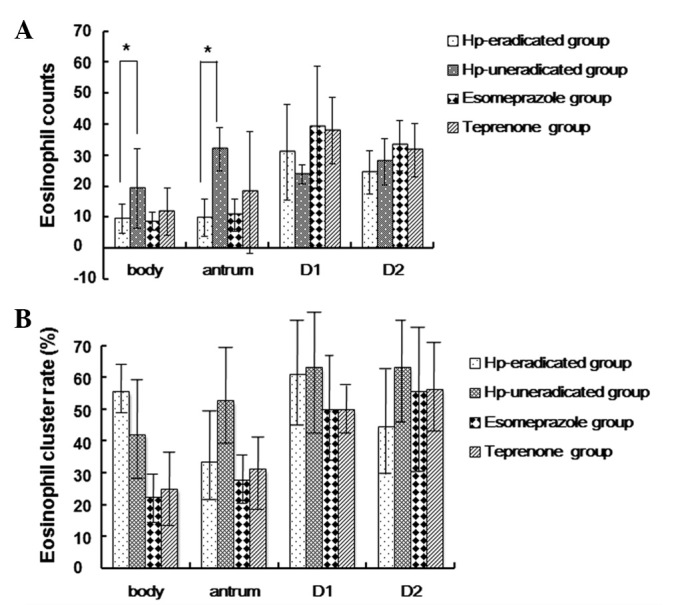
Eosinophil counts and cluster rate of 4 groups at week 6. (A) Eosinophil counts in the *H. pylori* (Hp)-eradicated (n=36), *H. pylori*-uneradicated (n=19), esomeprazole (n=18) and teprenone (n=16) groups. (B) Eosinophil cluster rate (%) calculated by χ^2^ analysis in the four groups. Results are shown as mean ± standard deviation (SD). ^*^P<0.05, single sample t-test. D1, duodenal bulb; D2, descending part of the duodenum.

Next, we investigated whether the commonly used drugs and *H. pylori* eradication treatment affect the gastroduodenal eosinophil counts and whether there is a correlation between symptom improvement and eosinophil counts. Compared with the *H. pylori*-uneradicated group, eosinophil counts in the antrum and body were significantly reduced in the *H. pylori*-eradicated group at week 6 (Fig. 3). In the duodenal bulb and the descending duodenum, the eosinophil counts of the *H. pylori*-eradicated group were not significantly different from those of the *H. pylori*-uneradicated groups at week 6.

The gastric and duodenal eosinophil counts of the *H. pylori*-uneradicated group did not present a significant reduction after week 6, while the gastric eosinophil counts of the *H. pylori*-eradicated group were reduced at week 6 compared with baseline; however, the duodenal eosinophil counts did not show a marked change between the two time points (Table III).

The eosinophil counts of the four gastroduodenal sites in the esomeprazole group were not statistically different from those of the teprenone group at week 6 (Fig. 3). Moreover, the gastric and duodenal eosinophil counts of the esomeprazole and teprenone groups were unchanged, respectively, between baseline and week 6 of treatment (Table IV), indicating that esomeprazole and teprenone are not effective for the treatment of eosinophilia.

### Eosinophil clusters

At baseline, compared with the *H. pylori*-negative group, the gastric eosinophil cluster rate was significantly increased in the *H. pylori*-positive group, whereas the duodenal eosinophil cluster rate of the *H. pylori*-positive group was not significantly different from that of the *H. pylori*-negative group (Table I). At week 6, the gastroduodenal eosinophil cluster rates were not significantly different between the *H. pylori*-eradicated and *H. pylori-*uneradicated groups (Fig. 3), or between the esomeprazole and teprenone groups (Table IV). The gastroduodenal eosinophil cluster rates did not show significant differences before and after treatment in the four groups (Tables III and IV). These results suggest that *H. pylori* infection upregulates gastroduodenal eosinophil clustering and that the eosinophil clusters are not reduced immediately after *H. pylori* eradication. Moreover, gastroduodenal eosinophil clusters are not affected by esomeprazole or teprenone treatment.

## Discussion

FD is a heterogeneous disorder characterized by the presence of recurrent or persistent symptoms originating in the gastroduodenal region without any organic, systemic or metabolic disease that is likely to explain the symptoms (24). The treatment of FD is based on the relief of symptoms rather than the treatment of abnormal pathophysiology (24–26).

In 2006, Rome III reformulated the FD classification method, which, to date, is the most recognized definition of FD. According to the Rome III criteria, FD must include one or more of the following symptoms: bothersome postprandial fullness, early satiation, epigastric pain and epigastric burning, with no evidence of structural disease for at least 3 months, with symptom onset at least 6 months before.

The role of *H. pylori* in FD is not fully understood. Currently, eradication treatment of *H. pylori* in FD continues to be controversial, even though a certain number of patients with FD appear to benefit from *H. pylori* eradication treatment (27). One study identified a tendency toward greater symptomatic benefit with *H. pylori* eradication therapy when compared with control treatment in patients with FD, particularly in an area with a high prevalence of infection (28). A study assessed the clinical course of FD during a long follow-up period of 7 years in a homogeneous sample of *H. pylori*-eradicated patients, which demonstrated that only a proportion of patients (10–50%) were symptom-free following eradication and at each 12-month evaluation, whereas other patients became symptomatic at different time points. FD symptoms were slightly improved following *H. pylori* eradication over a long period of time; however, a large percentage of these improved patients may experience FD symptoms again, even after a number of years of well-being after *H. pylori* eradication (29).

Our study identified that overall the symptom scores were not significantly different between the *H. pylori*-positive group and the *H. pylori*-negative group and the symptom scores of the *H. pylori*-eradicated group were not significantly different from those of the *H. pylori*-uneradicated group at baseline, week 2 and week 6 of treatment. These data suggest that *H. pylori* infection is not associated with the symptoms of FD and *H. pylori* eradication is not necessary when treating this disorder.

Eosinophils are effector cells that have immunomodulatory effects by releasing cytokines and presenting antigens. In animal models, eosinophils have been suggested to cause gastrointestinal dysmotility and impaired gastric relaxation, which lead to gastrointestinal symptoms, including abdominal pain and bloating. However, its role in human gastrointestinal diseases remains unclear (30). A striking, statistically significant increase of eosinophils was identified in *H. pylori*-infected gastric mucosa compared with *H. pylori*-negative gastritis with similar activity (31). Talley *et al* identified that gastric eosinophil numbers are greater in *H. pylori*-positive subjects, implicating that the infection may upregulate eosinophils (13). The concentration of ‘regulated on activation, normal T cell expressed and secreted’ (RANTES), a potent chemoattractant peptide for eosinophils, and the numbers of RANTES-positive cells were evaluated in the gastric mucosa from patients with *H. pylori*-positive chronic gastritis before and after *H. pylori* eradication and from *H. pylori*-negative healthy volunteers. The results revealed that RANTES protein concentration and RANTES-positive cells were significantly elevated in *H. pylori*-positive cases and remained high immediately after *H. pylori* eradication. The levels tended to decrease following *H. pylori* eradication but did not reach the level of *H. pylori*-negative cases, even at 24 months after *H. pylori* eradication. Therefore it appears to be an important mechanism of prolonged gastric mucosal immune response against *H. pylori* infection, even after *H. pylori* eradication (32).

Studies focusing on the correlation between *H. pylori* infection and duodenal eosinophilia are scarce. Talley *et al* identified that *H. pylori* infection has no significant relevance to duodenal eosinophilia (13). The current study identified that eosinophil counts in the gastric body and antrum were significantly increased in the *H. pylori*-positive group compared with the *H. pylori*-negative group, while eosinophil numbers in the duodenum were unchanged. The number of gastric eosinophil clusters was affected in the same manner. A similar tendency was observed between the *H. pylori*-eradicated and *H. pylori*-uneradicated groups. The results demonstrated that *H. pylori* infection upregulates gastric eosinophil counts but has no effect on duodenal eosinophil counts in patients with FD.

Moreover, the pathophysiology of FD is considered to be associated with duodenal motility disorders; however, its pathogenesis has not been fully elucidated. A previous study identified that the increased number eosinophils may be involved in the pathogenesis of FD. Talley *et al* reported that eosinophils in the upper gastrointestinal tract may be biomarkers for non-ulcer dyspepsia. Increased duodenal eosinophils may be the characteristic of a certain type of FD (13). It may be the case that eosinophils are a key link of neuroimmunoregulation in FD rather than the consequence.

The current therapy for FD is symptomatic and largely ineffective (24–26). Empiric acid suppression with PPIs is normally used in the treatment of FD. Generally, acid suppressive therapy using PPIs appears effective in patients whose predominant symptoms are epigastric pain or burning sensation (33,34). The role of acid and the presence of duodenal hypersensitivity to acid in FD patients remains controversial. Samsom *et al* reported that duodenal acid infusion induced nausea in a subset of FD patients but not in healthy controls, suggesting the presence of duodenal hypersensitivity to acid in FD patients (35). Duodenal acid perfusion (0.2 mol/l, 5 ml/min) for 15 min significantly exacerbated symptoms, including discomfort, bloating, nausea and epigastric burning in healthy subjects (36). In another study, no significant differences in dyspeptic symptom scores were observed before and after duodenal acid infusion and between duodenal saline and acid infusion (37).

Increased spontaneous duodenal acid exposure has also been identified in FD patients (38). One study demonstrated that reduced duodenal acid clearance plays a role in increasing duodenal acid exposure in FD patients (39). However, the mechanism remains unknown. The prolonged duodenal exposure to acid appears to be responsible for the generation of dyspeptic symptoms through the induction of gastric motor and sensory dysfunction (30).

*H. pylori* eradication in FD benefits a minority of cases but is worthwhile as the response may be maintained. Certain prokinetics may also be effective in the treatment of FD; patients with meal-related symptoms have the best response. Antidepressant therapy may also have a place in the management of difficult cases; however, adequate randomized controlled trials are unavailable (21). According to the study by Talley *et al*, targeted drugs that inhibit eosinophil production or eosinophil-derived products, including leukotriene-receptor antagonists, humanized monoclonal antibody against inter-leukin-5 and histamine 1 and 2 antagonists, may be effective for the treatment of FD (13).

In the current study, we identified that *H. pylori* infection increases gastric eosinophil counts, which may be induced by the upregulation of RANTES. The symptoms of FD improved following treatment with either esomeprazole or teprenone; however, no corresponding changes in duodenal eosinophil counts were observed. Moreover, our results demonstrated that symptom improvement resulted from treatment with esomeprazole but was not related to the clearance of *H. pylori*. In the duodenal bulb and the descending duodenum, the eosinophil counts of the *H. pylori*-eradicated group were not significantly different from those of the *H. pylori*-uneradicated group at week 6, suggesting that symptom improvement was also not associated with gastroduodenal eosinophil numbers.

In our study, esomeprazole, an acid suppressive drug, provided significant relief of symptoms in FD patients. The effect of esomeprazole on patients with FD may result from reduced duodenal acid exposure and corresponding reductions in sensitivity to gastric distension, impaired fundic accommodation to a meal and slow gastric emptying.

The commonly used therapies for gastrointestinal disorders in China are PPIs, domperidone and teprenone. Teprenone is often used for gastritis and gastric ulcers and its pharmacological effects include anti-ulcer effects, increase of gastric mucus, improvement of gastric mucosal blood flow, protection of the gastric mucosa and induction of heat shock protein genesis. Studies on the effect of teprenone on FD are rare. Previously published data revealed that teprenone tended to improve only gastric stasis (GSS) and that only 52% of patients treated with teprenone favored their medication (40).

In the current study, teprenone was used for the treatment of *H. pylori*-negative patients with FD and their symptoms improved at week 2 compared with those at baseline, but not at week 6. The mechanism remains unknown, however, we hypothesize that the improvement of gastric mucosal blood flow and increase of gastric mucus may help to reduce the gastroduodenal sensitivity to stimulus. The protective effect is, however, limited and temporary. Esomeprazole was shown to be more effective than teprenone in the treatment of FD.

As the follow-up period of the present study was relatively short and the FD patients were not divided into subtypes of postprandial distress syndrome (PDS) and epigastric pain syndrome (EPS), further studies are required to investigate the mechanisms involved. The results demonstrated that duodenal eosinophil number is not associated with *H. pylori* infection or the esomeprazole or teprenone treatment of FD. Moreover, esomeprazole appears to be more effective than teprenone and *H. pylori* eradication is not necessary in the treatment of FD. The effect of a PPI on FD observed in the current study suggest that hypersensitivity to acid and/or increased duodenal acid exposure are associated with the pathophysiology of FD.

## Figures and Tables

**Table I.** Symptom scores and eosinophil counts in FD patients at baseline.

Group	Symptom scores	Eosinophil counts (mean ± SD)				Cluster rate of eosinophil (%)			
		Body	Antrum	D1	D2	Body	Antrum	D1	D2
Hp-positive (n=84)	4.48±1.91	26.33±18.8	20.35±16.2	31.62±15.2	28.83±15.6	32.14	20.24	40.48	38.10
Hp-negative (n=72)	4.69±1.81	10.92±11.2	9.63±9.60	33.06±19.3	31.31±15.9	11.11	5.56	37.5	36.11
P-value^a^	0.48	0.00	0.00	0.60	0.33	0.00	0.01	0.69	0.79

FD, functional dyspepsia; D1, duodenal bulb; D2, descending part of the duodenum; Hp, *Helicobactor pylori;* SD, standard deviation.

a Comparison between Hp-positive and Hp-negative groups.

**Table II.** Symptom scores of the four groups before and after treatment (mean ± SD).

Groups	Baseline	Week 2	Week 6
Hp-eradicated group (n=58)	5.21±1.42	1.93±1.33^a^	0.71±0.75^b^,^c^
Hp-uneradicated group (n=26)	4.65±1.97	1.73±1.33^a^	0.77±0.96^b^
P-value^d^	0.14	0.53	0.76
Esomeprazole group (n=36)	4.92±1.57	1.33±0.57^a^	0.67±0.62^b^
Teprenone group (n=36)	4.53±1.87	1.67±1.15^a^	3.67±3.21
P-value^e^	0.34	0.12	0.00

a P<0.05, week 2 vs. baseline;

b P<0.05, week 6 vs. baseline;

c P<0.05, week 6 vs. week 2.

d Hp-eradicated group vs. Hp-uneradicated group;

e esomeprazole group vs. teprenone group. SD, standard deviation; Hp, *Helicobactor pylori*.

**Table III.** Eosinophil counts and cluster rates of the Hp-eradicated group (group A) and Hp-uneradicated group (group B) at baseline and week 6.

	Eosinophil counts (mean ± SD)				Cluster rate of eosinophils (%)			
	Body	Antrum	D1	D2	Body	Antrum	D1	D2
Baseline of group A (n=58)	20.86±12.11	14.36±13.65	27.60±11.12	27.07±13.99	39.66	20.70	43.10	31.03
Week 6 of group A (n=36)	9.36±4.78	9.78±6.02	30.92±15.47	24.36±7.08	55.56	33.33	61.11	44.44
P-value^a^	0.00	0.06	0.23	0.28	0.13	0.17	0.09	0.19
Baseline of group B (n=26)	25.00±19.57	23.65±16.63	28.65±14.03	29.08±9.03	34.62	38.46	57.69	57.69
Week 6 of group B (n=19)	19.26±12.77	31.74±7.01	23.74±3.11	27.74±7.55	42.10	52.63	63.16	63.16
P-value^a^	0.27	0.053	0.14	0.60	0.49	0.34	0.71	0.71

Hp, *Helicobactor pylori*; SD, standard deviation; D1, duodenal bulb; D2, descending part of the duodenum.

a Baseline vs. week 6 of treatment.

**Table IV.** Eosinophil counts and cluster rate of the esomeprazole (group C) and teprenone groups (group D) at baseline and week 6.

	Eosinophil counts (mean ± SD)				Cluster rate of eosinophils (%)			
	Body	Antrum	D1	D2	Body	Antrum	D1	D2
Baseline of group C (n=36)	7.78±7.66	7.61±5.00	30.83±12.56	31.47±14.21	19.44	33.33	50.00	63.89
Week 6 of group C (n=18)	8.50±2.89	10.5±5.20	39.00±19.63	33.00±8.04	22.22	27.77	50.00	55.56
P-value^a^	0.70	0.053	0.07	0.67	0.81	0.68	1.00	0.13
Baseline of group D (n=36)	9.56±5.62	12.14±4.07	35.56±24.05	31.11±17.89	22.22	27.78	55.56	58.33
Week 6 of group D (n=16)	11.69±7.63	18.00±19.64	37.81±10.74	31.50±8.60	25.00	31.25	50.00	56.25
P-value^a^	0.27	0.09	0.72	0.99	0.83	0.80	0.71	0.89

SD, standard deviation; D1, duodenal bulb; D2, descending part of the duodenum.

a Baseline vs. week 6 of treatment.
